# The effect of initial teaching on evaluation of left ventricular volumes by cardiovascular magnetic resonance imaging: comparison between complete and intermediate beginners and experienced observers

**DOI:** 10.1186/s12880-017-0197-5

**Published:** 2017-05-17

**Authors:** Erik Hedström, Masaki Ishida, Alvaro Sepúlveda-Martínez, Daniel Ryd, Johannes Sperling, Henrik Engblom, Eike Nagel

**Affiliations:** 1 Division of Imaging Sciences and Biomedical Engineering, King’s College London, London, UK; 2 BHF Centre of Research Excellence and NIHR Biomedical Research Centre at Guy’s and St Thomas’ NHS Foundation Trusts and King’s College London, London, UK; 3 Skane University Hospital, Department of Clinical Sciences Lund, Clinical Physiology, Lund University, Lund, Sweden; 4 Skane University Hospital, Department of Clinical Sciences Lund, Diagnostic Radiology, Lund University, Lund, Sweden; 5 Fetal i + D Fetal Medicine Research Center, BCNatal - Barcelona Center for Maternal-Fetal and Neonatal Medicine (Hospital Clínic and Hospital Sant Joan de Deu), Institut Clínic de Ginecologia, Obstetricia i Neonatologia, Institut d’Investigacions Biomèdiques August Pi i Sunyer, Universitat de Barcelona, and Centre for Biomedical Research on Rare Diseases (CIBER-ER), Barcelona, Spain; 6 Institute for Experimental and Translational Cardiovascular Imaging, Goethe University, Frankfurt/Main and DZHK (German Centre for Cardiovascular Research, Standort RheinMain), Frankfurt, Germany

**Keywords:** CMR, Teaching, Cine, Function, Beginners

## Abstract

**Background:**

High reproducibility and low intra- and interobserver variability are important strengths of cardiac magnetic resonance (CMR). In clinical practice a significant learning curve may however be observed. Basic CMR courses offer an average of 1.4 h dedicated to lecturing and demonstrating left ventricular (LV) function analysis. The purpose of this study was to evaluate the effect of initial teaching on complete and intermediate beginners’ quantitative measurements of LV volumes and function by CMR.

**Methods:**

Standard clinical cine CMR sequences were acquired in 15 patients. Five observers (two complete beginners, one intermediate, two experienced) measured LV volumes. Before initial evaluation beginners read the SCMR guidelines on CMR analysis. After initial evaluation, beginners participated in a two-hour teaching session including cases and hands-on training, representative for most basic CMR courses, after which it is uncertain to what extent different centres provide continued teaching and feedback in-house. Dice Similarity Coefficient (DSC) assessed delineations. Agreement, accuracy, precision, repeatability and reliability were assessed by Bland-Altman, coefficient of variation, and intraclass correlation coefficient methods.

**Results:**

Endocardial DSC improved after teaching (+0.14 ± 0.17;*p* < 0.001) for complete beginners. Low intraobserver variability was found before and after teaching, however with wide limits of agreement. Beginners underestimated volumes by up to 44 ml (EDV), 27 ml (ESV) and overestimated LVM by up to 53 g before teaching, improving to an underestimation of up to 9 ml (EDV), 7 ml (ESV) and an overestimation of up to 30 g (LVM) after teaching. For the intermediate beginner, however, accuracy was quite high already before teaching.

**Conclusions:**

Initial teaching to complete beginners increases accuracy for assessment of LV volumes, however with high bias and low precision even after standardised teaching as offered in most basic CMR courses. Even though the intermediate beginner showed quite high accuracy already before teaching, precision did generally not improve after standardised teaching. To maintain CMR as a technique known for high accuracy and reproducibility and low intra- and inter-observer variability for quantitative measurements, internationally standardised training should be encouraged including high-quality feedback mechanisms. Objective measurements of training methods, training duration and, above all, quality of assessments are required.

## Background

High reproducibility and low intra- and interobserver variability of cardiac magnetic resonance (CMR) assessments of quantitative data are important strengths of this technique in clinical practice and in its role as an endpoint in research studies. Although CMR has been demonstrated to be a highly accurate imaging technique for measuring left ventricular (LV) volumes [1], it has been noted that a significant learning curve may be observed in clinical practice. In basic CMR courses, as based on nine international course schedules available online, an average of 1.4 h dedicated to lecturing and demonstrating LV function analysis is offered. It is however unclear to what extent different centres provide continued teaching and feedback in-house to beginners to further improve accuracy and precision in CMR measurements after initial teaching. There are currently no international criteria to measure the quality of “sufficient” training for the individual based on accuracy and precision, but rather a time frame and number of cases performed, as presented in the guidelines for training and accreditation of CMR in Europe [2]. It is the senior authors’ experience that whereas some centres have a reference population that should be accurately and precisely measured by beginners with feedback from experienced observers as part of in-house training, other centres may have no particular in-house training or validation of CMR beginners’ measurements before they are allowed to report clinically or actively participate in research studies.

Previous teaching studies have shown improvement for measuring LV parameters in healthy volunteers and patients after teaching for observers with up to 2 years previous training [3, 4], indicating the importance of continued teaching and feedback. The impact of short standardised teaching on CMR measurements of LV volumes and function in patients has however not been assessed for complete beginners, although the limited teaching time offered in basic courses may be hypothesised to not lead to a major change in measurements due to the complexity of CMR. Further, there is an increasing number of physicians in want of CMR training leading to a potential move from training in high-volume centres to lower-volume centres. It is thus important to have knowledge of the impact of basic training also on complete beginners’ measurements, to maintain CMR as a technique known for its high accuracy and reproducibility and low intra- and inter-observer variability.

The aim of the current study was therefore to evaluate the effect of initial teaching on complete and intermediate beginners’ quantitative measurements of LV volumes and function in patients, and to compare these with experienced observers’ measurements.

## Methods

### Study population

The local ethics board approved the study and written informed consent was obtained from all participants. Fifteen patients (13 male, median age 68 years, range 52–82 years) were included. The patients were prospectively and randomly selected from the clinical population, with a history of symptoms representative of stable angina and known or suspected coronary artery disease (CAD). Patients with an estimated glomerular filtration rate < 30 ml/min/1.73 m^2^ or with contraindications to CMR were excluded, according to clinical routine.

### Image acquisition

Cardiac magnetic resonance imaging was performed using standard clinical techniques according to recognised SCMR international guidelines [5]. All CMR examinations were performed on a 3 T MR scanner (Achieva, Philips, Best, the Netherlands), using a 32-channel phased-array receiver coil. The study examination included routinely acquired cine short- and long-axis balanced steady state free precession (bSSFP) images after administration of a gadolinium-based contrast agent (gadobutrol, Berlin-Wedding, Schering, Germany).

### Image analysis

All analyses were performed in accordance with the SCMR guidelines [6]. The cine images were analysed using the freely available software Segment (version 2.0; Medviso AB, Lund, Sweden) for quantitative measurements [7]. Results were blinded to all observers between assessments and between observers. In total five observers (two complete beginners, one intermediate beginner, and two experienced observers) independently evaluated the images. The two complete beginners were medical students (B1, B2), without previous experience in LV CMR delineation. The intermediate beginner (IB) had experience from foetal cardiac ultrasound as an obstetrician, and had delineated 20 CMR cases without formal training before participating in this study. The two experienced CMR level III-certified observers with 15 years CMR experience had trained at the same centre (E1, E2). All observers independently measured LV volumes including LV mass twice, at least one week apart. In the case of beginners, assessments were also repeated twice after the teaching sessions.

Before the initial assessment, the beginners read the standardised SCMR description on how to analyse LV volumes and function [6]. After the first assessment, the three beginners attended a one-hour lecture including cases and discussions on how to delineate LV endo- and epicardial borders in bSSFP cine images, with a total of seven attendees. The beginners also attended a one-hour hands-on teaching session on how to delineate endo- and epicardial borders for LV volumes and function. As only the three beginners participating in the current study attended this hands-on session, much attention was given to interaction for direct feedback from the teacher. The CMR level III-accredited observer E1 gave both teaching sessions.

### Statistical analyses

The software R (version 3.0.2) was used for all statistical analyses [8]. Inter-observer variability between beginner and expert observers was based on each observer’s first measurement; in the case of beginners the first measurements both before and after teaching, respectively, to avoid impact of the repeat measurements on precision and accuracy calculations after teaching. Beginners’ accuracy and precision was calculated as bias and 95% limits of agreement (i.e. ±1.96 standard deviations) between each beginner and E1 [9]. The coefficient of variation (CV) was calculated for further assessment of agreement and was defined as the SD of differences between the respective measurements divided by their mean and expressed in %. For reliability, the intraclass correlation coefficient (ICC) was computed using the R irr v0.84 package as a one-way single consistency score as an index of intra- and inter-rater reliability for quantitative data [10], for inter-rater reliability based on each observer’s first measurement. Negative ICC values may occur when within-groups variance exceeds the between-groups variance, and were marked * in Table 1.

**Table 1:** Agreement, accuracy, precision, repeatability and reliability for LV volumes, function and mass measurements

EDV (ml)				Coefficient of variability (%)			Limits of agreement			ICC		
		1st	2nd	Intra- observer	Inter-observer	Precision	Intra-observer	Inter-observer	Accuracy/precision	Intra-observer	Inter-observer	Accuracy
B1	pre	120 ± 24	120 ± 29	11.2	-	9.0	1 ± 26	-	−44 ± 25	0.880	-	0.177
	post	155 ± 28	158 ± 31	6.1	-	5.8	3 ± 19	-	−9 ± 18	0.944	-	0.910
B2	pre	127 ± 27	135 ± 29	6.8	-	9.8	8 ± 17	-	−37 ± 28	0.916	-	0.357
	post	165 ± 31	166 ± 35	7.5	-	7.8	2 ± 24	-	1 ± 25	0.932	-	0.921
IB	pre	158 ± 32	154 ± 30	7.0	-	6.5	−4 ± 21	-	−6 ± 20	0.934	-	0.931
	post	162 ± 29	172 ± 34	6.7	-	5.8	10 ± 22	-	−2 ± 19	0.898	-	0.952
E1		164 ± 32	164 ± 32	1.7	-	-	0 ± 5	-	-	0.996	-	-
E2		160 ± 32	161 ± 32	2.5	2.5	-	0 ± 8	−4 ± 8	-	0.993	0.986	-
ESV (ml)				Coefficient of variability (%)			Limits of agreement			ICC		
		1st	2nd	Intra- observer	Inter-observer	Precision	Intra-observer	Inter-observer	Accuracy/precision	Intra-observer	Inter-observer	Accuracy
B1	pre	52 ± 27	49 ± 29	20.0	-	16.5	−3 ± 20	-	−27 ± 21	0.933	-	0.601
	post	72 ± 35	82 ± 35	23.4	-	15.7	10 ± 36	-	−7 ± 24	0.836	-	0.916
B2	pre	57 ± 30	52 ± 26	27.3	-	19.6	−4 ± 29	-	−23 ± 26	0.858	-	0.692
	post	77 ± 40	78 ± 40	14.7	-	16.0	0 ± 22	-	−2 ± 25	0.961	-	0.942
IB	pre	80 ± 34	81 ± 36	9.2	-	10.2	1 ± 15	-	0 ± 16	0.979	-	0.972
	post	80 ± 33	85 ± 36	8.8	-	6.9	5 ± 14	-	0 ± 11	0.969	-	0.986
E1		80 ± 33	80 ± 33	1.9	-	-	0 ± 3	-	-	0.999	-	-
E2		81 ± 33	80 ± 33	3.6	4.4	-	0 ± 6	1 ± 7	-	0.996	0.994	-
SV (ml)				Coefficient of variability (%)			Limits of agreement			ICC		
		1st	2nd	Intra- observer	Inter-observer	Precision	Intra-observer	Inter-observer	Accuracy/precision	Intra-observer	Inter-observer	Accuracy
B1	pre	67 ± 11	71 ± 13	21.2	-	16.6	4 ± 29	-	−17 ± 25	0.243	-	^a^
	post	83 ± 16	76 ± 9	23.0	-	20.6	−7 ± 36	-	−2 ± 33	^a^	-	0.251
B2	pre	70 ± 16	83 ± 16	25.5	-	21.6	12 ± 38	-	−14 ± 34	0.138	-	^a^
	post	88 ± 14	89 ± 15	14.5	-	12.9	1 ± 25	-	3 ± 22	0.621	-	0.565
IB	pre	78 ± 13	73 ± 11	15.7	-	13.3	−5 ± 23	-	−7 ± 21	0.471	-	0.467
	post	82 ± 10	87 ± 10	14.1	-	12.0	5 ± 23	-	−2 ± 20	0.252	-	0.504
E1		84 ± 10	84 ± 10	3.9	-	-	0 ± 6	-	-	0.953	-	-
E2		80 ± 10	81 ± 12	5.1	6.8	-	1 ± 8	−4 ± 11	-	0.933	0.775	-
EF (%)				Coefficient of variability (%)			Limits of agreement			ICC		
		1st	2nd	Intra- observer	Inter-observer	Precision	Intra-observer	Inter-observer	Accuracy/precision	Intra-observer	Inter-observer	Accuracy
B1	pre	58 ± 13	61 ± 15	15.8	-	14.1	3 ± 19	-	5 ± 16	0.756	-	0.678
	post	55 ± 15	50 ± 10	23.9	-	18.9	−5 ± 25	-	2 ± 20	0.468	-	0.683
B2	pre	57 ± 15	62 ± 12	22.0	-	19.0	5 ± 26	-	4 ± 21	0.503	-	0.613
	post	55 ± 13	55 ± 14	12.9	-	11.2	0 ± 14	-	2 ± 12	0.870	-	0.857
IB	pre	51 ± 11	49 ± 11	10.9	-	10.3	−2 ± 11	-	−2 ± 10	0.878	-	0.855
	post	52 ± 10	52 ± 10	8.3	-	7.7	0 ± 9	-	−1 ± 8	0.913	-	0.920
E1		53 ± 10	53 ± 10	2.4	-	-	0 ± 2	-	-	0.992	-	-
E2		51 ± 10	52 ± 10	3.6	5.1	-	0 ± 4	−2 ± 5	-	0.984	0.954	-
LVM (g)				Coefficient of variability (%)			Limits of agreement			ICC		
		1st	2nd	Intra- observer	Inter-observer	Precision	Intra-observer	Inter-observer	Accuracy/precision	Intra-observer	Inter-observer	Accuracy
B1	pre	162 ± 23	160 ± 24	5.8	-	9.5	−2 ± 18	-	53 ± 25	0.921	-	^a^
	post	140 ± 31	131 ± 20	15.5	-	18.2	−9 ± 41	-	30 ± 44	0.645	-	0.123
B2	pre	158 ± 26	154 ± 29	7.0	-	10.2	−4 ± 22	-	49 ± 26	0.915	-	^a^
	post	134 ± 21	115 ± 18	7.6	-	9.1	−19 ± 19	-	24 ± 21	0.546	-	0.262
IB	pre	100 ± 17	107 ± 16	5.8	-	7.7	8 ± 12	-	−10 ± 16	0.835	-	0.690
	post	111 ± 16	105 ± 15	6.1	-	6.8	−6 ± 13	-	1 ± 15	0.855	-	0.884
E1		110 ± 14	111 ± 15	3.0	-	-	1 ± 6	-	-	0.972	-	-
E2		111 ± 16	110 ± 15	3.4	2.9	-	−1 ± 7	1 ± 6	-	0.969	0.979	-

*LV* left ventricle, *EDV* end-diastolic volume, *ESV* end-systolic volume, *SV* stroke volume, *EF* ejection fraction, *LVM* LV mass, Limits of agreement: bias ± 1.96SD. *ICC* Intra-class correlation coefficient. *B1-B2* complete beginners, *IB* intermediate beginner, *E1-E2* expert observers. *Pre* before teaching; *post* after teaching.^a^ negative ICC value (see Statistics section for further explanation). Inter-observer between experts. Accuracy and precision between beginners and E1

The Dice Similarity Coefficient (DSC) was calculated between beginners and E1 for endo- and epicardial delineations, respectively, on a slice-by-slice basis for both end-diastole and end-systole. Only slices in which both the respective beginner and E1 had performed delineations were included in the averages presented in Table 2, and grouped for complete cases and for basal, midventricular and apical thirds of the left ventricle for readability. This matched slice-by-slice DSC was performed to avoid falsely reported averaged imperfect matches due to delineations in basal or apical slices where observers may differ in opinion on which slice to include, and thus these slices would risk to lower the overall score despite perfect matches in other slices. The number of cases where the beginners and expert chose to define basal and apical slices differently is instead presented separately. The DSC formula was based on two times the volume of the intersection of the respective two regions divided by the sum of the regional volumes, with DSC = 0 if the regions did not overlap at all and DSC = 1 if the regions overlapped perfectly [11]. As the D’Agostino & Pearson omnibus normality test showed non-Gaussian distribution, the Mann–Whitney test was performed to assess differences before and after teaching, with a *p* value < 0.05 indicating statistically significant differences.

**Table 2:** Dice Similarity Coefficients (DSC) reported as mean ± SD before and after teaching between the complete beginners (B1, B2) and the intermediate beginner (IB) vs. the expert observer (E1)

	DSC B1		DSC B2		DSC IB	
	Before	After	Before	After	Before	After
End-diastole endocardium	0.83 ± 0.08	0.93 ± 0.07^***^	0.84 ± 0.10	0.93 ± 0.08^***^	0.94 ± 0.08	0.95 ± 0.06^ns^
End-systole endocardium	0.70 ± 0.13	0.90 ± 0.08^***^	0.68 ± 0.17	0.84 ± 0.15^***^	0.90 ± 0.09	0.92 ± 0.06^ns^
End-diastole epicardium	0.93 ± 0.07	0.92 ± 0.10^ns^	0.92 ± 0.12	0.92 ± 0.12^ns^	0.94 ± 0.11	0.94 ± 0.12^ns^
End-systole epicardium	0.90 ± 0.12	0.91 ± 0.11^ns^	0.89 ± 0.15	0.88 ± 0.15^ns^	0.92 ± 0.15	0.93 ± 0.09^**^
Basal end-diastole endocardium	0.88 ± 0.06	0.94 ± 0.03^***^	0.90 ± 0.05	0.95 ± 0.03^***^	0.95 ± 0.04	0.96 ± 0.03^ns^
Midventricular end-diastole endocardium	0.80 ± 0.06	0.95 ± 0.02^***^	0.84 ± 0.06	0.96 ± 0.02^***^	0.96 ± 0.02	0.97 ± 0.01^ns^
Apical end-diastole endocardium	0.81 ± 0.09	0.88 ± 0.11^***^	0.77 ± 0.14	0.87 ± 0.12^***^	0.89 ± 0.15	0.91 ± 0.09^ns^
Basal end-diastole epicardium	0.93 ± 0.08	0.91 ± 0.14^ns^	0.91 ± 0.17	0.94 ± 0.11^ns^	0.96 ± 0.03	0.95 ± 0.11^ns^
Midventricular end-diastole epicardium	0.96 ± 0.03	0.96 ± 0.02^ns^	0.96 ± 0.02	0.96 ± 0.02^ns^	0.97 ± 0.01	0.97 ± 0.01^ns^
Apical end-diastole epicardium	0.90 ± 0.08	0.88 ± 0.09^ns^	0.89 ± 0.11	0.84 ± 0.17^ns^	0.87 ± 0.19	0.89 ± 0.16^ns^
Basal end-systole endocardium	0.76 ± 0.12	0.91 ± 0.05^***^	0.78 ± 0.13	0.90 ± 0.06^***^	0.94 ± 0.03	0.94 ± 0.03^ns^
Midventricular end-systole endocardium	0.64 ± 0.12	0.92 ± 0.03^***^	0.62 ± 0.14	0.85 ± 0.10^***^	0.92 ± 0.04	0.94 ± 0.03^**^
Apical end-systole endocardium	0.66 ± 0.13	0.86 ± 0.11^***^	0.61 ± 0.19	0.76 ± 0.21^***^	0.84 ± 0.15	0.88 ± 0.09^ns^
Basal end-systole epicardium	0.89 ± 0.16	0.92 ± 0.12^*^	0.88 ± 0.21	0.88 ± 0.21^ns^	0.94 ± 0.13	0.94 ± 0.08^ns^
Midventricular end-systole epicardium	0.95 ± 0.02	0.95 ± 0.03^ns^	0.94 ± 0.02	0.94 ± 0.03^ns^	0.97 ± 0.02	0.96 ± 0.01^***^
Apical end-systole epicardium	0.87 ± 0.11	0.87 ± 0.11^ns^	0.86 ± 0.11	0.85 ± 0.11^ns^	0.85 ± 0.21	0.90 ± 0.12^ns^

For a perfect match in delineations DSC = 1 whereas 0 indicates no overlap in delineations. Basal, midventricular and apical denote slices comprising one third of the longitudinal left ventricle for end-diastole and end-systole, respectively. ^ns^non-significant, *p < 0.05, **p < 0.01, ***p < 0.001 after teaching vs. before teaching

## Results

The set of CMR images were representative of a general clinical population, including infarcted regions, thinning of myocardium and hypertrophy, and a range of hypo-/a- and dyskinesia. Images ranged from good overall and diagnostic quality to images affected by artefacts. Intra- and inter-observer variability and agreement, accuracy, precision, repeatability and reliability for beginners and experts are displayed in Table 1. Compared with both intra- and inter-observer data for experienced observers, beginners generally showed higher variability, lower agreement and lower repeatability, as could be expected.

### Endo- and epicardial delineations

The DSC showed an improvement in overall delineations for complete beginners for both end-diastolic and end-systolic endocardial delineations, whereas epicardial delineations did not improve significantly after teaching, except for basal end-systole for B1 (Table 2). When comparing the DSC values between basal, midventricular and apical thirds of the longitudinal length of the LV it should be noted that basal but even more so apical DSC values are expected to be lower than midventricular values as the generally smaller areas yield inherently lower DSC. Values should therefore be compared between observers before and after teaching for the separate LV thirds, rather than between separate thirds. The beginners generally improved their definition of which basal and apical slices to include after teaching, and correspondingly missed fewer basal and apical slices after teaching (Table 3). Noteworthy, the beginners did not include more basal or apical slices than the expert for any case.

**Table 3:** Number of basal and apical slices not delineated by the complete beginners (B1, B2) and the intermediate beginner (IB) at end-diastole and end-systole before and after teaching, compared with expert observer E1

	B1		B2		IB	
	Before	After	Before	After	Before	After
Basal end-diastole endocardial	2	2	5	3	4	3
Basal end-systole endocardial	7	1	12	7	6	5
Basal end-diastole epicardial	1	0	3	2	4	2
Basal end-systole epicardial	4	0	9	4	5	3
Apical end-diastole endocardial	6	2	4	4	6	3
Apical end-systole endocardial	8	5	5	3	8	3
Apical end-diastole epicardial	0	1	1	0	0	0
Apical end-systole epicardial	0	0	0	0	1	0

**Fig. 1:**
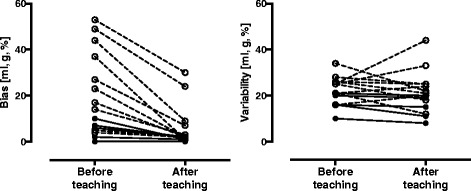
Representative cases of basal (*top row*), midventricular (*middle row*) and apical (*bottom row*) slices in end-diastole (*upper left*) and end-systole (*upper right*) before teaching, and corresponding images after teaching for end-diastole (*lower left*) and end-systole (*lower right*) for the two complete beginners (B1 and B2) and the intermediate beginner (IB). Delineations by expert observer 1 (E1) are included for comparison

Representative delineations before (Fig. 1 top row) and after (Fig. 1 bottom row) teaching indicate typical “delineation errors” before teaching, such as exclusion of papillary muscle from blood pool (albeit not incorrect per se, but generally not performed for clinical delineation) by the complete beginners and lack of apical endocardial delineation by all beginners, but also the more challenging case of the basal delineation in end-systole where the experts followed the guidelines and extended the delineation to the aortic valve leaflets. It may be argued that variability in delineation of the basal slice may decrease if only ventricular muscle and corresponding blood pool were to be delineated instead of extending the delineations to the leaflets. This was however not particularly investigated in the current study, as the standardised SCMR guidelines were the chosen basis for delineations. Further, it was noted that these beginners’ errors were corrected after teaching, and also that the general circular shape of the ventricle was more closely followed (Fig. 1 bottom row).

### Intra-observer measures

For EDV, intra-observer variability for beginners showed improvement after teaching for B1 as indicated by both decreased CV (11.2 vs. 6.1), decreased limits of agreement (26 vs. 19) and increased ICC (0.880 vs. 0.944). For both B2 and IB, however, there was no clear change in intra-observer variability after teaching.

For ESV, a different pattern emerged in which B2 improved in intra-observer variability, whereas B1 rather showed an increased variability and IB again showed no major difference after teaching. The mean values for volume measurements for each beginner’s repeated measures before and after teaching were similar, showing quite low bias and overall high ICC values (Table 1). The limits of agreement were however overall comparably wide also after teaching.

For LVM, bias did not show a clear improvement after teaching and again limits of agreement were wide and ICC showed a sharp decrease for intra-observer reliability after teaching for both B1 and B2 (Table 1).

### Inter-observer, accuracy and precision measures

**Fig. 2:**
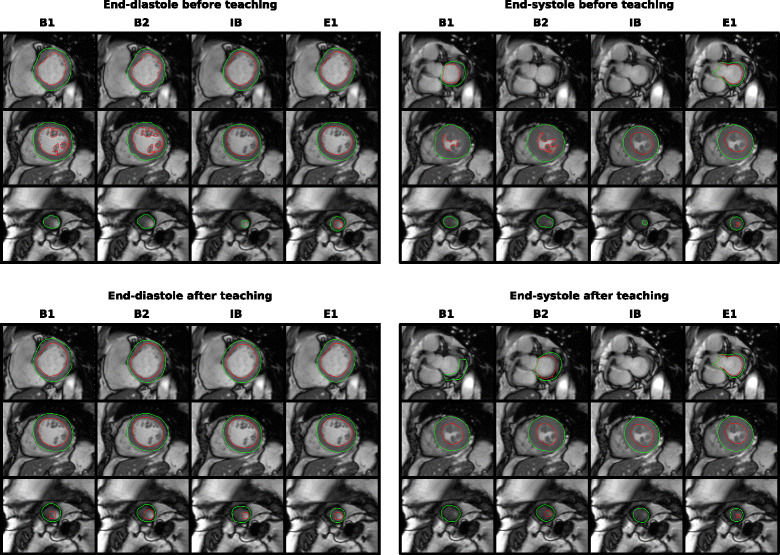
Minimal teaching and training in left ventricular delineation, as in the current study, may lead to a direction of increased accuracy, i.e. decreased bias (*left*) but not necessarily higher precision, i.e. lower variability (*right*) for beginners. The graphs show pooled left ventricular data in absolute values for both complete beginners (*open circles*, *dashed lines*) and the intermediate beginner (*solid circles* and *lines*) before and after teaching

Accuracy and precision before and after teaching are shown in Fig. 2. Precision as quantified by CV for beginners’ measurements of EDV and ESV showed improvement after teaching, but to a generally small extent, which is supported by a small change also in precision measured as limits of agreement. The limits of agreement were quite wide also after teaching. The beginners underestimated measured volumes by up to 44 ml (EDV), 27 ml (ESV) and overestimated LVM by up to 53 g before teaching, improving to an underestimation of up to 9 ml (EDV), 7 ml (ESV) and an overestimation of up to 30 g (LVM) after teaching. It is important to note that ejection fraction (EF) showed a generally high accuracy for all beginners also before teaching, indicating the imperative in stating measured volumes, i.e. EDV and ESV, and not EF alone as it hides potential delineation errors and incorrect volume measurements. Thus, the measure of EF cannot be used as a proxy for accurate and precise measurement of volumes.

The ICC showed generally lower accuracy compared with intra-observer reliability for the two complete beginners, and showed negative values in some cases, indicating within-groups variance exceeding between-groups variance (marked * in Table 1). More importantly, accuracy as measured by ICC increased strongly after teaching. For the intermediate beginner, however, accuracy measured as bias and as ICC for EDV and ESV was high already before teaching, particularly so for ESV. The intermediate beginner on the other hand showed an increase in accuracy for LVM after teaching.

Trends of improvement in intra-observer reliability after teaching did not necessarily indicate improved accuracy after teaching, and trends of decrease in intra-observer reliability were also found in cases with increased accuracy (Table 1). This indicates that the beginners improved in assessment compared with the expert in some cases, but at a cost of decreased reliability in repeated assessments, important to acknowledge in continued training.

### LVM at end-diastole and end-systole

Left ventricular mass measured in diastole and systole can be used as an internal control for measurement errors as it should be similar throughout the cardiac cycle with minimum theoretical changes related to myocardial blood volume. For the experienced observer this difference was −0.1 ± 0.8 g, whereas the beginners generally showed higher bias and variability, both before (IB: −3.1 ± 6.9 g; B1: −0.1 ± 18.1 g; B2: −8.2 ± 10.6 g) and after teaching (IB: −5.2 ± 6.4 g; B1: 2.2 ± 13.5 g; B2: −17.9 ± 12.6 g).

## Discussion

This CMR study shows that initial teaching to complete beginners increases accuracy for assessment of left ventricular volume measurements, however with high bias and low precision even after teaching. Even though the intermediate beginner showed quite high accuracy already before teaching, precision did generally not improve after standardised teaching as offered in most basic CMR courses. Thus, a two-hour teaching session is insufficient to allow complete and intermediate beginners to adequately evaluate basic CMR studies for left ventricular volumes and function.

When comparing accuracy and precision in the current study with previous studies some important points in how to plan teaching sessions need to be considered. In the previous teaching study by Karamitsos et al. [3] the beginner observers had up to two years previous training and were taught how to delineate CMR images also before their two-month study training period. We showed generally lower accuracy and precision for complete beginners, as expected, but similar in parts for the intermediate beginner in the current study. In their study, however, the most basal and apical slices were excluded from analysis, which inherently may increase accuracy and precision as compared with experienced observers when excluding these more challenging slices, although at a cost of accuracy and precision vs. true volumes. Compared with the study by Groth et al. [4], accuracy was lower but precision similar for EDV and ESV in the current study for the complete beginners, despite that the beginners in the previous study by Groth et al. all had a minimum of six months training before participating in the teaching study. This is also indicated by that the intermediate beginner in the current study showed similar accuracy and precision for EDV, whereas he measured ESV more accurately than the beginners in the previous study. Both the current study and the study by Groth et al. show improvement in accuracy but not precision after teaching. Altogether, this shows the importance of not focusing on time and number of cases only for deciding on whether a beginner is sufficiently trained.

In general, beginners’ underestimation of EDV and ESV and overestimation of LVM, as in the current study, can be related to systematic errors in defining which basal slice to include for measurements and incorrect inclusion of papillary muscle and trabeculation in mid-ventricular slices. As an example, both B1 and B2 in the current study assumed before teaching that papillary muscle and trabeculation should be included as myocardium, which is consistent with the more pronounced observed underestimation of EDV and ESV, and corresponding overestimation of LVM for these observers. This also stresses the importance of reporting measured EDV and ESV, and not EF alone as EF showed low bias (5%) despite the large underestimation of EDV (−44 and −37 ml) and ESV (−27 and −23 ml) before teaching, and reporting only on EF would thereby be misleading.

Previous studies showing improvement in CMR assessment after teaching included observers with six months to two years previous CMR experience [4], or previous cardiovascular or imaging knowledge [3]. In the current study, the intermediate observer functioned as a bridge between previous studies and the complete beginners in the current study. Together with the previous studies, the current study indicates both the value of previous experience, regardless of whether this is in CMR, other imaging or cardiovascular anatomy knowledge, and the need for intensive training. Most importantly, it is obvious that physicians may not receive appropriate initial training as improvement is found also two years after initial training as shown previously [4]. An initial short teaching session as given in most basic CMR courses is thus not sufficient and the individual differences between complete CMR beginners, the intermediate beginner and observers already with up to two years previous CMR experience are large. Therefore both adequate initial teaching and continuous experience including systematic training and above all adequate feedback in-house is needed for development of satisfactory skills in CMR evaluation. Even though CMR level II and III definitions include a time frame/number of cases for training; 3 months/150 cases and 12 months/300 cases, respectively, there are currently no criteria to measure the quality of “sufficient” training for the individual trainee [2].

In the current study both accuracy and precision of LV volumes were low for complete beginners without previous theoretical or clinical experience in CMR, irrespective of acceptable intra-observer variability. As shown by the intermediate beginner already before teaching, however, already a basic background in cardiac physiology and CMR analysis resulted in high reliability for both EDV (ICC: 0.177 and 0.357 vs. 0.931 for B1, B2 and IB, respectively) and ESV (ICC: 0.601 and 0.692 vs. 0.972 for B1, B2 and IB, respectively) as compared with expert observers. Whereas complete beginners demonstrated a clear improvement in volume measurements of both EDV and ESV after teaching, the intermediate beginner showed only minor changes in accuracy, being high already before teaching. However, the intermediate beginner did not improve in precision for EDV and ESV after teaching, indicating reproducibility errors. These reproducibility errors may be related to a combination of excluded basal slices, mismatch of delineation vs. trabeculation and for ESV difficulties in defining the proper lumen area considering that papillary muscle is included. All of which can be handled by focussed training after high-quality feedback with specific advice and recommendations on how to use the adjacent slices and different time frames for guidance.

For LVM, measurements rely on delineation of both endo- and epicardial borders. In the current study accuracy for LVM was low compared with accuracy for EDV and ESV, indicating challenges in delineating the epicardial border in particular. This is also supported by the higher reliability ICC values for EDV and ESV between beginners and experts. The DSC results may seem contra intuitive to this as no large differences in epicardial delineations between beginners and expert were shown, but this can be related to the inherent weakness of the DSC method as also relatively large area differences between observers for the relatively large epicardial delineations only have small impact on DSC values, i.e. a larger variability for epicardial delineations as DSC values are similar to (the smaller area of the) endocardial delineations and thus myocardial volumes and LVM may be different even though not indicated by DSC as such. Even though teaching improved beginners’ assessments of LVM, the low accuracy and precision also after teaching are unacceptable for clinical reports and research studies. Further teaching with adequate feedback from experienced observers in-house is required to maintain CMR as an accurate and precise method. The reason for why the epicardial borders may be challenging could be associated with for instance chemical shift artefacts, which if not recognised will lead to inconsistent delineations with impact on variability, as noted in the performed by the complete beginners. Teaching led the beginners to understand the chemical shift artefacts and how to delineate the epicardial border in these cases. Together with properly clinically delineated papillary muscle and trabeculation after teaching, the LVM accuracy improved, however insufficient for clinical reporting or research studies. The decreased precision for LVM by B1, however, may be related to that this observer found the endocardial border more difficult to define, particularly in end-systole where papillary muscle and trabeculation may be challenging to differ from the actual myocardial wall. As this observer challenge was identified, focussed continued training may quickly help this observer to improve. This was also indicated by that the last measurement rendered an accuracy and precision of 21 ± 22 g, i.e. an improvement compared with previous measurements, without the interaction of the expert or continued teaching.

Altogether, the beginners in the current study showed trends of both increased and decreased reliability after teaching. This may indicate individual differences between observers, and points to the fact that individualised feedback is crucial when structuring training.

For training purposes it may also be useful to perform delineations in patients without shunts or valvular disease and provide the differences in stroke volumes (SV) between the left and right ventricles as a feedback mechanism. Similarly, utilising the diastolic and systolic LVM measurements as internal validation is considered to lower variability and increase accuracy in LVM determination. As this is generally not part of basic courses, the teaching did not include this recommendation. The results also show that the beginners did not consider LVM as an internal control and this single addition may further improve measurements.

The use of medical students as complete beginners may seem exaggerated, as most physicians may have had previous cardiac imaging experience before turning towards CMR. However, considering that CMR research and cardiac delineations are sometimes being performed also by engineers with potentially little background in cardiac anatomy and imaging experience, and by students without formal CMR training, we considered it valuable to assess delineations of complete beginners also representative for these groups. Finally, a higher number of observers may increase the power of the study. Study of the outcome of continued training of the beginners in the current study, or lack of training, would indicate the accuracy over time in centres with and without formalised continued training, and most importantly presence or absence of high-quality feedback mechanisms.

It has recently been shown that also experienced observers from different centres may vary in measured volumes, particularly for basal and apical slices but also dependent on small and consistent disparities throughout the short-axis stack [12]. A strategy to decrease inter-observer variability could be to move towards more automated delineation by employing computer algorithms, as routinely performed in nuclear medicine [13]. This has however been challenging in CMR even though recent improvements of automatic algorithms show promise for the future [14]. The use of automatic algorithms without previous training in delineation however leads to lower overall delineation competence. The observer may not be sufficiently knowledgeable to delineate particularly challenging cases where the automatic algorithm may fail. Basic and thorough training is thus necessary before applying the use of automatic delineation software to maintain CMR as a technique known for its high accuracy and reproducibility and low intra- and inter-observer variability for quantitative measurements.

### Limitations

Image contrast between blood pool and myocardium was slightly reduced as contrast agent had been administered before acquisition of cine images. This may have impact on delineation of endocardial borders for volume measurements. Also, the anatomical changes related to presence of pathology found in some of the included patients may render delineation more complex. The study population is however also because of these limitations representative of consecutive clinical patients and the results may therefore be considered adequate for clinical imaging and research studies. The number of observers may be considered small, however equivalent to previous teaching and observer variability studies, although the current study does not provide the highest number of observers among these studies. As the observers in previous studies represented more heterogeneous groups it may however be argued that the number in the current study is satisfactory.

## Conclusions

This CMR study shows that initial teaching to complete beginners increases accuracy for assessment of left ventricular volume measurements, however with high bias and low precision even after teaching. Even though the intermediate beginner showed quite high accuracy already before teaching, precision did generally not improve after standardised teaching as offered in most basic CMR courses. To maintain CMR as a technique known for its high accuracy and reproducibility and low intra- and inter-observer variability for quantitative measurements, internationally standardised training should be encouraged including high-quality feedback mechanisms. Objective measurements of training methods, training duration and, above all, quality of assessments are required.

## Abbreviations

B1: Beginner 1
B2: Beginner 2
bSSFP: Balanced steady state free precession
CAD: Coronary artery disease
CMR: Cardiovascular magnetic resonance
CV: Coefficient of variation
DSC: Dice similarity coefficient
E1: Expert observer 1
EDV: End-diastolic volume
EF: Ejection fraction
ESV: End-systolic volume
IB: Intermediate beginner
ICC: Intraclass correlation coefficient
LV: Left ventricle
LVM: Left ventricular mass
SCMR: Society for Cardiovascular Magnetic Resonance
SD: Standard deviation
SV: Stroke volume

## References

[1] Lorenz CH, Walker ES, Morgan VL, Klein SS, Graham TP (1999). Normal human right and left ventricular mass, systolic function, and gender differences by cine magnetic resonance imaging. J Cardiovasc Magn Reson.

[2] Plein S, Schulz-Menger J, Almeida A, Mahrholdt H, Rademakers F, Pennell D (2011). Training and accreditation in cardiovascular magnetic resonance in Europe: a position statement of the working group on cardiovascular magnetic resonance of the European Society of Cardiology. Eur Heart J.

[3] Karamitsos TD, Hudsmith LE, Selvanayagam JB, Neubauer S, Francis JM (2007). Operator induced variability in left ventricular measurements with cardiovascular magnetic resonance is improved after training. J Cardiovasc Magn Reson.

[4] Groth M, Muellerleile K, Klink T, Säring D, Halaj S, Folwarski G (2012). Improved agreement between experienced and inexperienced observers using a standardized evaluation protocol for cardiac volumetry and infarct size measurement. Fortschr Röntgenstr.

[5] Kramer CM, Barkhausen JR, Flamm SD, Kim RJ, Nagel E (2013). Standardized cardiovascular magnetic resonance (CMR) protocols 2013 update. J Cardiovasc Magn Reson.

[6] Schulz-Menger J, Bluemke DA, Bremerich J, Flamm SD, Fogel MA, Friedrich MG (2013). Standardized image interpretation and post processing in cardiovascular magnetic resonance: society for cardiovascular magnetic resonance (SCMR) board of trustees task force on standardized post processing. J Cardiovasc Magn Reson.

[7] Heiberg E, Sjögren J, Ugander M, Carlsson M, Engblom H, Arheden H (2010). Design and validation of Segment--freely available software for cardiovascular image analysis. BMC Med Imaging.

[8] R Core Team. R: A language and environment for statistical computing. Vienna: R Foundation for Statistical Computing; 2013. https://www.R-project.org/. Accessed 31 Oct 2016.

[9] Bland JM, Altman DG (1986). Statistical methods for assessing agreement between two methods of clinical measurement. Lancet.

[10] Shrout PE, Fleiss JL (1979). Intraclass correlations: uses in assessing rater reliability. Psychol Bull.

[11] Dice LR (1945). Measures of the amount of ecologic association between species. Ecology.

[12] Suinesiaputra A, Bluemke DA, Cowan BR, Friedrich MG, Kramer CM, Kwong R, et al. Quantification of LV function and mass by cardiovascular magnetic resonance: multi-center variability and consensus contours. J Cardiovasc Magn Reson. 2015;17–63. 10.1186/s12968-015-0170-9 PMC4517503 26215273

[13] Germano G, Kiat H, Kavanagh PB, Moriel M, Mazzanti M, Su HT (1995). Automatic quantification of ejection fraction from gated myocardial perfusion SPECT. J Nucl Med.

[14] Tufvesson J, Hedström E, Steding-Ehrenborg K, Carlsson M, Arheden H, Heiberg E (2015). Validation and development of a New automatic algorithm for time-resolved segmentation of the left ventricle in magnetic resonance imaging. Biomed Res Int.

