# Correction to: The effect of initial teaching on evaluation of left ventricular volumes by cardiovascular magnetic resonance imaging: comparison between complete and intermediate beginners and experienced observers

**DOI:** 10.1186/s12880-022-00771-z

**Published:** 2022-03-10

**Authors:** Erik Hedström, Masaki Ishida, Alvaro Sepúlveda-Martínez, Daniel Ryd, Johannes Sperling, Henrik Engblom, Eike Nagel

**Affiliations:** 1 Division of Imaging Sciences and Biomedical Engineering, King’s College London, London, UK; 2 BHF Centre of Research Excellence and NIHR Biomedical Research Centre at Guy’s and St Thomas’ NHS Foundation Trusts and King’s College London, London, UK; 3 Skane University Hospital, Department of Clinical Sciences Lund, Clinical Physiology, Lund University, Lund, Sweden; 4 Skane University Hospital, Department of Clinical Sciences Lund, Diagnostic Radiology, Lund University, Lund, Sweden; 5 Fetal I + D Fetal Medicine Research Center, BCNatal - Barcelona Center for Maternal-Fetal and Neonatal Medicine (Hospital Clínic and Hospital Sant Joan de Deu), Institut Clínic de Ginecologia, Obstetricia i Neonatologia, Institut d’Investigacions Biomèdiques August Pi I Sunyer, Universitat de Barcelona, and Centre for Biomedical Research On Rare Diseases (CIBER-ER), Barcelona, Spain; 6 Institute for Experimental and Translational Cardiovascular Imaging, Goethe University, Frankfurt/Main and DZHK (German Centre for Cardiovascular Research, Standort RheinMain), Frankfurt, Germany

## Correction to: BMC Medical Imaging (2017) 17:33 10.1186/s12880-017-0197-5

Since the publication of the original article [1], the fourth author has changed his name from “Daniel Salehi” to “Daniel Ryd”.

The correct name is included in the author list of this Correction and has already been updated in the original article.
